# Latent profiles of adherence to lymphedema prevention behaviors and associated factors among breast cancer patients after surgery: a cross-sectional study

**DOI:** 10.3389/fpubh.2026.1972630

**Published:** 2026-09-21

**Authors:** Ziqi Guo, Hang Liu, Yanyan Wang, Ying Li, Wei Liu, Qing Zhao, Jing Li, Xing Li

**Affiliations:** 1 Department of Breast Surgery, the First Affiliated Hospital of Zhengzhou University, Zhengzhou, China; 2 Department of Urology, the First Affiliated Hospital of Zhengzhou University, Zhengzhou, China; 3 Department of Ophthalmology, the First Affiliated Hospital of Zhengzhou University, Zhengzhou, China; 4 Office of Retiree Affairs, the First Affiliated Hospital of Zhengzhou University, Zhengzhou, China

**Keywords:** adherence, breast cancer, interoceptive sensitivity, latent profile analysis, lymphedema, prevention behavior, self-regulatory fatigue

## Abstract

**Background:**

Breast cancer-related lymphedema (BCRL) is a common complication after breast cancer surgery and can have lasting effects on physical function and quality of life. Prevention behaviors are an important component of BCRL risk reduction, yet patients do not necessarily engage in these behaviors in the same way. Previous studies have largely assessed adherence at the overall level, providing limited insight into differences in behavioral patterns among postoperative patients. A person-centered approach may therefore help identify distinct adherence profiles and provide a basis for targeted prevention and rehabilitation.

**Methods:**

A cross-sectional study was conducted from July 2025 to March 2026 among 612 postoperative breast cancer patients recruited from the breast surgery outpatient clinic of a tertiary hospital in Zhengzhou, China, using convenience sampling. Participants completed the Adherence to Lymphedema Prevention Behaviors Scale for Breast Cancer Survivors, the Multidimensional Assessment of Interoceptive Awareness, Version 2 (MAIA-2), the Self-Regulatory Fatigue Scale (SRF-S), and a general information questionnaire. Latent profile analysis (LPA) was used to identify distinct adherence profiles, followed by multinomial logistic regression to examine factors associated with profile membership.

**Results:**

Latent profile analysis identified three distinct profiles of adherence to lymphedema prevention behaviors among postoperative breast cancer patients: global low-adherence profile (*n* = 82, 13.4%), the moderate adherence with skin-care deficit profile (*n* = 110, 18.0%), and the active prevention profile (*n* = 420, 68.6%). Multinomial logistic regression analysis showed that age, tumor stage, history of chemotherapy or radiotherapy, self-regulatory fatigue, and interoceptive sensitivity were significant factors associated with latent profile membership (*p* < 0.05).

**Conclusion:**

Adherence to lymphedema prevention behaviors showed substantial heterogeneity among postoperative breast cancer patients. Dynamic assessment of adherence should be incorporated into clinical care, with stratified management and individualized interventions tailored to the behavioral characteristics of different profiles. Particular attention should be given to patients with lower adherence to strengthen prevention behaviors and support postoperative rehabilitation.

## 1 Introduction

Breast cancer is the most frequently diagnosed malignancy and a leading cause of cancer-related mortality among women worldwide, with an estimated 3 million new cases projected annually by 2040 (1, 2). In China, breast cancer similarly ranks as the most common female malignancy, accounting for approximately 416,000 new cases per year and representing 18.4% of the global disease burden (3). Advances in multimodal treatment have substantially improved breast cancer survival, with 5-year survival rates now exceeding 80% (4). As survival continues to improve, the focus of breast cancer care has increasingly shifted from disease control to long-term recovery and functional well-being.

Surgery remains an essential component of comprehensive treatment for early-stage breast cancer. As diagnostic and treatment techniques have continued to advance, patient survival has improved markedly, yet a range of postoperative complications continue to substantially affect patients’ long-term quality of life (5). Breast cancer-related lymphedema (BCRL) is a common chronic complication following breast cancer surgery (6) and is characterized by a progressive, irreversible course. BCRL may develop in the early postoperative period and can persist or gradually worsen over time (7), producing limb swelling, pain, restricted movement, and functional impairment that add to patients’ psychological burden and markedly compromise their quality of life and social functioning (8). Because no effective cure for BCRL currently exists (9), strengthening its prevention and early management is of considerable importance for supporting patients’ long-term functional recovery and improving their quality of life (10).

Existing research indicates that adherence to lymphedema prevention behavior among postoperative breast cancer patients remains suboptimal overall and varies considerably between individuals (11) a pattern shaped by both treatment-related factors and individual behavioral characteristics (12). Prior studies have examined several factors contributing to psychosocial distress in breast cancer patients, including negative emotions such as anxiety and depression (13), illness perception (14), financial strain (15), and social support (8). However, most existing work has focused on long-term self-management after BCRL has already developed, while comparatively little attention has been given to prevention behavior among patients in the early postoperative period.

The Health Action Process Approach (HAPA) posits that the development and maintenance of health behaviors are dynamic processes shaped by multiple factors, including risk perception, behavioral intention, self-efficacy, and self-regulation (16). HAPA emphasizes that health behavior requires not only the formation of behavioral intentions but also sustained self-regulation during the volitional phase, including action planning, action control, and maintenance of self-efficacy (16). To date, no published study has examined heterogeneity in adherence to lymphedema prevention behaviors among patients after breast cancer surgery from a person-centered perspective. Therefore, guided by the HAPA, this study used latent profile analysis (LPA) to identify distinct profiles of adherence to lymphedema prevention behaviors among patients after breast cancer surgery and to examine the factors associated with profile membership. Previous research has shown that self-regulatory processes are closely associated with health behavior maintenance (8), suggesting that differences in the psychological resources available for self-regulation may be relevant to patients’ ability to consistently perform lymphedema prevention behaviors. In addition, HAPA identifies risk perception as an important factor in the motivational phase, while interoceptive sensitivity reflects an individual’s ability to perceive and interpret internal bodily signals. Previous studies have linked interoceptive sensitivity to self-management behaviors among patients with breast cancer (17), suggesting that sensitivity to bodily signals may be relevant to patients’ awareness of potential health risks and engagement in preventive behaviors. Therefore, examining self-regulatory fatigue and interoceptive sensitivity from the perspective of the HAPA framework may provide a more comprehensive understanding of individual differences in adherence to lymphedema prevention behaviors. Previous studies have shown that sociodemographic characteristics, such as age and educational attainment, as well as interoceptive sensitivity and self-regulation, are associated with lymphedema prevention behaviors among patients with breast cancer (8, 17). However, previous research has primarily examined these factors at the population level and has paid limited attention to heterogeneity in adherence to lymphedema prevention behaviors among individual patients. Identifying distinct subgroups of patients based on their patterns of adherence and examining the factors associated with these subgroups may facilitate more targeted patient stratification and individualized nursing care.

patterns of adherence, this study aimed to provide a basis for the precise identification of patients with different behavioral profiles and the development of stratified, individualized nursing interventions.

## 2 Subjects and methods

### 2.1 Study design and participants

A cross-sectional study was conducted using convenience sampling to recruit patients who had undergone breast cancer surgery and attended follow-up visits at the Department of Breast Surgery, the First Affiliated Hospital of Zhengzhou University, between July 2025 and March 2026. The inclusion criteria were as follows: (1) age ≥18 years; (2) meeting the diagnostic criteria for breast cancer specified in the Guidelines and Specifications for the Diagnosis and Treatment of Breast Cancer of the Chinese Anti-Cancer Association (2026 edition) (18); (3) having completed surgery, chemotherapy, and/or radiotherapy 1 week to 5 years previously and being in the rehabilitation stage during the disease consolidation period (19); and (4) having sufficient comprehension and verbal communication abilities and providing informed consent to participate in the study. The exclusion criteria were as follows: (1) severe physical illness or a psychiatric disorder; and (2) current participation in another study.

According to the Kendall rule of thumb (20), the required sample size should be 10–15 times the number of independent variables. Eleven independent variables were included in the present study, resulting in an estimated sample size of 110–165 participants. After allowing for a 20% rate of invalid questionnaires, the required sample size was estimated to be 138–206 participants. To ensure the stability and accuracy of parameter estimation in the latent profile analysis and multinomial logistic regression models, 612 patients after breast cancer surgery were ultimately included in the study.

### 2.2 Study procedure

Two nursing postgraduate students who had received standardized training served as investigators. Before the survey, the investigators explained the study purpose and procedures, instructions for completing the questionnaires, and measures for protecting participants’ privacy to the patients and their family members. Written informed consent was obtained from each participant before the survey was initiated. Questionnaires were collected and checked on site, and any missing responses were immediately clarified with the participant and completed. After data collection, all questionnaires were independently reviewed and entered into the database by two investigators. A total of 630 questionnaires were distributed, of which 612 were valid, yielding a valid response rate of 97.14%.

### 2.3 Variables and instruments

#### 2.3.1 General information questionnaire

A general information questionnaire was developed by the researchers to collect information on age, educational attainment, employment status, type of health insurance, place of residence, monthly per capita household income, tumor stage, type of surgery, receipt of chemotherapy and/or radiotherapy, whether the affected limb was the dominant limb, and whether axillary lymph node dissection had been performed.

#### 2.3.2 Adherence to lymphedema prevention behaviors scale for breast Cancer patients in the rehabilitation stage

The Adherence to Lymphedema Prevention Behaviors Scale for Breast Cancer Patients in the Rehabilitation Stage was developed by our research team (21) to assess adherence to BCRL prevention behaviors among patients after breast cancer surgery. The scale comprises five dimensions and 30 items: protection of the affected limb, skin care, appropriate functional exercise, self-monitoring and symptom recognition, and healthy lifestyle. A 5-point Likert scale was used, with responses ranging from “never” to “always,” scored from 1 to 5, respectively, with higher scores indicating better adherence. A score index was also calculated to standardize adherence to prevention behaviors according to the following formula: Score index = (actual score / maximum possible score) × 100%. Based on the score index, adherence to lymphedema prevention behaviors was categorized into three levels: low (<40%), moderate (40–80%), and high (>80%). The scale demonstrated good internal consistency, with a Cronbach’s α coefficient of 0.901, and the content validity index ranged from 0.825 to 1.000.

#### 2.3.3 Multidimensional assessment of interoceptive awareness, version 2, MAIA-2

The Multidimensional Assessment of Interoceptive Awareness, Version 2 (MAIA-2), developed by Mehling et al. (22) and translated into Chinese by Teng et al. (23), was used to assess the level of interoceptive sensitivity. The scale comprises seven dimensions and 31 items: Not-Distracting, Not-Worrying, Attention Regulation, Emotional Awareness, Self-Regulation, Body Listening, and Trust. A 6-point Likert scale was used, ranging from “never” to “always,” scored from 0 to 5, respectively. Higher scores indicate greater interoceptive sensitivity. The Cronbach’s α coefficient for the overall scale was 0.822, with coefficients ranging from 0.656 to 0.830 across the dimensions. The scale has a systematic structure and comprehensive coverage of interoceptive dimensions and is suitable for use in Chinese populations. It has also been widely applied in breast cancer research.

#### 2.3.4 Self-regulatory fatigue scale, SRF-S

The Self-Regulatory Fatigue Scale (SRF-S), developed by Nes et al. (24) and translated into Chinese by Wang et al. (25), was used to assess the level of self-regulatory fatigue. The scale consists of 16 items across three dimensions: cognitive control, emotional control, and behavioral control. A 5-point Likert scale was used, with responses ranging from “strongly disagree” to “strongly agree,” scored from 1 to 5, respectively. Some items were reverse-scored, with responses from “strongly disagree” to “strongly agree” scored from 5 to 1, respectively. The total score ranges from 16 to 80, with higher scores indicating greater self-regulatory fatigue. The scale demonstrated good internal consistency, with a Cronbach’s α coefficient of 0.84.

### 2.4 Statistical analysis

Statistical analyses were performed using R software (version 4.3.2). Normally distributed continuous variables are reported as mean ± standard deviation; non-normally distributed variables are reported as median (interquartile range). Categorical variables are described using frequencies and percentages. Kruskal–Wallis tests were used to compare continuous variables across groups, and chi-square or Fisher’s exact tests were applied to categorical variables, as appropriate.

LPA was conducted using the mclust package in R, with the five dimension-level scores of the adherence scale serving as manifest indicators. Models with one to five profiles were fitted sequentially and compared using the Akaike Information Criterion (AIC), Bayesian Information Criterion (BIC), sample-size-adjusted BIC (aBIC), Entropy, Lo–Mendell–Rubin likelihood ratio test (LMRT), and bootstrapped likelihood ratio test (BLRT). Lower AIC, BIC, and aBIC values indicate better model fit; Entropy values closer to 1 indicate higher classification accuracy. Significant LMRT and BLRT results (*p* < 0.05) indicate that a k-profile model provides a significantly better fit than the (k–1)-profile model. The optimal model was selected by integrating all of these criteria alongside clinical interpretability. Variables considered for inclusion in the multinomial logistic regression model were selected based on previous literature, clinical relevance, and findings from univariate analyses. Multinomial logistic regression was then used to identify factors associated with profile membership, with a significance level of *α* = 0.05.

### 2.5 Ethical considerations

This study was conducted in accordance with the ethical standards of the institution and was approved by the Ethics Committee of the First Affiliated Hospital of Zhengzhou University (Approval No. 2024-KY-0299-002). All participants provided written informed consent before participating in the study. The survey did not collect personally identifiable information, and no clinical interventions were performed. The study was considered to involve no more than minimal risk to participants.

## 3 Results

### 3.1 Scores for adherence to lymphedema prevention behaviors, self-regulatory fatigue, and interoceptive sensitivity among patients after breast cancer surgery

The median score for adherence to lymphedema prevention behaviors among patients after breast cancer surgery was 108 (99, 116), with a score index of 72%. The median scores for the individual dimensions were 18 (15, 20) for skin care, 21 (19, 24) for protection of the affected limb, 21 (19, 23) for appropriate functional exercise, 29 (26, 27) for healthy lifestyle, and 18 (16, 20) for self-monitoring and symptom recognition. The median score for self-regulatory fatigue was 29 (25, 44), while the median score for interoceptive sensitivity was 105 (87, 110).

### 3.2 General characteristics of the participants

Among the 612 patients after breast cancer surgery, those aged <40 years constituted the largest age group, comprising 254 patients (41.50%). Educational attainment was relatively evenly distributed across junior college or above, technical secondary school/high school, and junior high school or below, accounting for 35.62, 34.80, and 29.58%, respectively. In terms of employment status, farming and employment were the two most common categories, accounting for 38.56 and 38.24%, respectively. The New Rural Cooperative Medical Scheme was the most common type of health insurance, accounting for 39.87% of the participants. A monthly per capita household income of < RMB 3,000 was most common, accounting for 40.36%.

Regarding disease characteristics, 337 patients (55.07%) had stage II disease. The most commonly performed surgical procedures were modified radical mastectomy (44.77%) and radical mastectomy (39.54%). Axillary lymph node dissection had been performed in 348 patients (56.86%), and the majority of patients (82.35%) had received chemotherapy or radiotherapy. The affected limb was the dominant limb in 55.39% of the participants.

### 3.3 Latent profile analysis of adherence to lymphedema prevention behaviors and profile identification

Latent profile analysis was conducted using the scores of the five dimensions of the Adherence to Lymphedema Prevention Behaviors Scale as observed indicators. Models with one to five profiles were sequentially fitted, and the results are presented in Supplementary Table 1. From the one-profile to the three-profile models, the AIC, BIC, and aBIC values decreased continuously and reached local minima in the three-profile model. When the number of profiles was further increased to four and five, the AIC, BIC, and aBIC values increased substantially, suggesting potential overfitting or deterioration in model fit. The LMRT and BLRT results showed that the *p* values for the two-profile and three-profile models were both <0.001, indicating that the addition of a profile significantly improved model fit compared with the preceding model. The entropy value of the three-profile model was **0.941**, indicating a high level of classification accuracy and clear separation between profiles. The profile probabilities were **0.134, 0.180, and 0.686**, respectively, with the smallest profile accounting for 13.4% of the sample and no profile containing an excessively small proportion of participants. Considering the information criteria, statistical significance of the model comparison tests, classification quality, and clinical interpretability, the **three-profile model was selected as the best-fitting model**.

The characteristics of the three profiles across the observed indicators are shown in Figure 1. Based on the score patterns across the five dimensions of adherence to lymphedema prevention behaviors, the profiles were named as follows:

**Figure 1:**
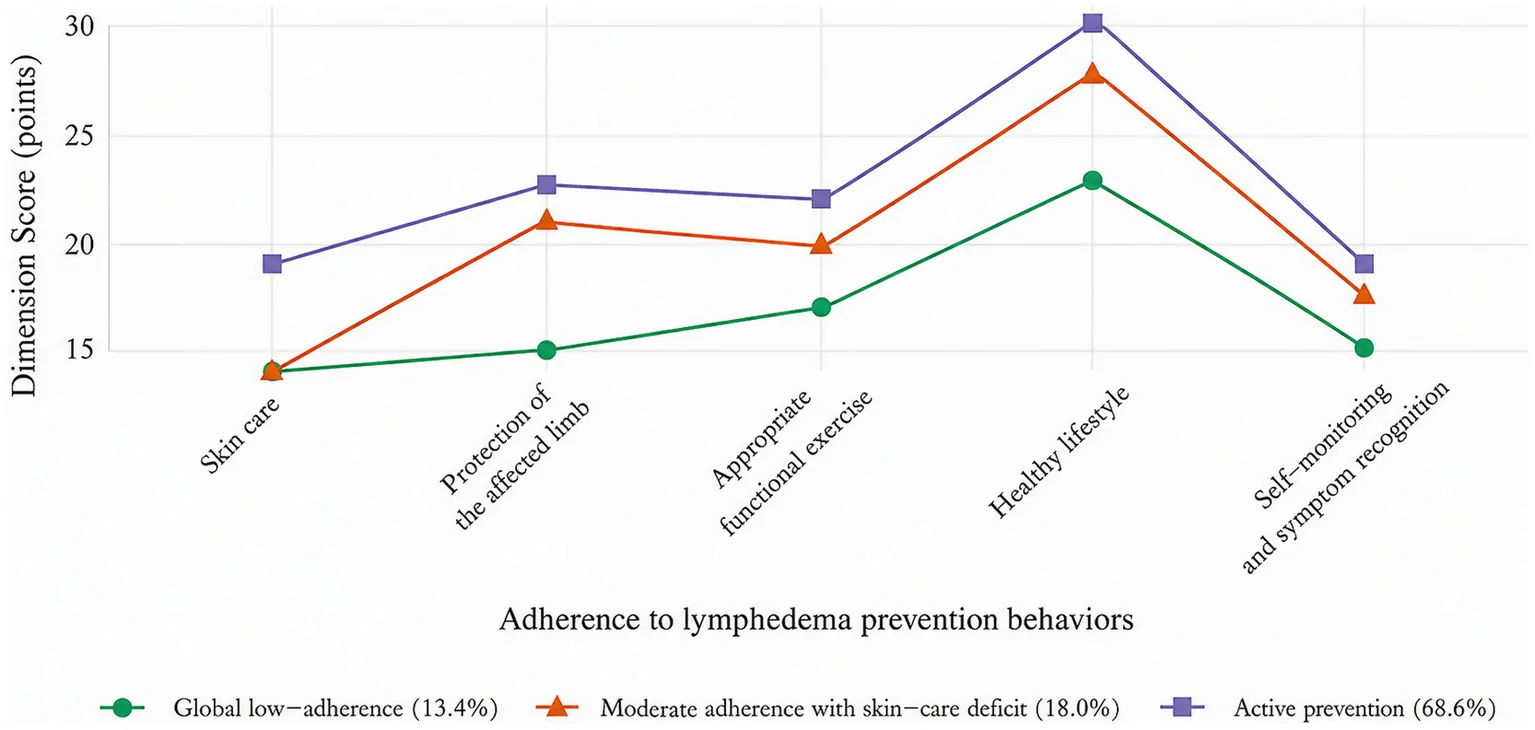
Latent profiles of adherence to lymphedema prevention behaviors among patients with breast cancer after surgery.

Profile 1: Global Low-Adherence (13.4%). Patients in this profile had the lowest scores across all five dimensions of adherence to lymphedema prevention behaviors among the three profiles, indicating an overall pattern of low engagement and neglect of postoperative preventive and rehabilitation behaviors.

Profile 2: Moderate Adherence with Skin-Care Deficit (18.0%). Patients in this profile showed moderate scores across the overall behavioral indicators, but their scores for skin care were relatively low and were close to those of the Global Low-Adherence profile. Therefore, this profile was characterized as moderate adherence with a specific deficit in skin-care behaviors.

Profile 3: Active Prevention (68.6%). Patients in this profile demonstrated consistently high levels of adherence across the behavioral dimensions and actively engaged in postoperative self-care and rehabilitation behaviors. This profile was therefore characterized as Active Prevention.

### 3.4 Univariate analysis of factors associated with latent profiles of adherence to lymphedema prevention behaviors

The general characteristics of the participants and the univariate analysis of latent profiles of adherence to lymphedema prevention behaviors are shown in Supplementary Table 2.

### 3.5 Multinomial logistic regression analysis of latent profiles of adherence to lymphedema prevention behaviors

The three latent profiles of adherence to lymphedema prevention behaviors identified by latent profile analysis were used as the dependent variable, with Profile 2 (Moderate Adherence with Skin-Care Deficit) and Profile 3 (Active Prevention) coded as 2 and 3, respectively, and Profile 1 (Global Low-Adherence) used as the reference category (coded as 1). Variables were selected for the multinomial logistic regression model based on previous literature, clinical relevance, and univariate analysis, including age, employment status, medical insurance, average monthly per-capita income, tumor stage, type of surgery, axillary lymph node dissection, chemotherapy/radiotherapy history, dominant hand involvement of the affected limb, self-regulatory fatigue, and interoceptive sensitivity. Continuous variables were entered into the model as their original values. Categorical variables were coded as follows: age (<40 years = 1, 40–49 years = 2, ≥50 years = 3); employment status (farming = 1, employed = 2, other = 3); type of health insurance (New Rural Cooperative Medical Scheme = 1, resident medical insurance = 2, employee medical insurance = 3); monthly per capita household income (<RMB 3,000 = 1, RMB 3,000–5,000 = 2, >RMB 5,000 = 3); tumor stage (stage I = 1, stage II = 2, stage III or above = 3); surgical type (radical mastectomy = 1, modified radical mastectomy = 2, breast-conserving surgery = 3); axillary lymph node dissection (yes = 1, no = 2); chemotherapy and/or radiotherapy (yes = 1, no = 2); and affected limb being the dominant limb (yes = 1, no = 2). The results of the multinomial logistic regression analysis are presented in Supplementary Table 3.

## 4 Discussion

### 4.1 Heterogeneity in adherence to lymphedema prevention behaviors among patients after breast cancer surgery

The present study found that the total score for adherence to lymphedema prevention behaviors among patients after breast cancer surgery was 108 (99, 116), with a score index of 72%, indicating a moderate overall level of adherence. This finding is generally consistent with previous studies on BCRL prevention behaviors in China (8, 28). The findings may reflect the increasing implementation of standardized breast cancer treatment, transitional care, and health education, alongside patients’ recognition of the importance of lymphedema prevention and adoption of corresponding health management behaviors in daily life. However, some patients may still have insufficient awareness of lymphedema risk and inadequate knowledge regarding prevention strategies. Moreover, maintaining prevention behaviors over time, such as protection of the affected limb, functional exercise, and health monitoring, may be challenging, which may affect long-term adherence (26). Therefore, continuous management of lymphedema risk after breast cancer surgery warrants continued attention in clinical care. Individualized health education, regular follow-up, and behavioral support may be relevant to patients’ risk awareness and self-management capacity and may be considered when developing strategies for the long-term maintenance of prevention behaviors.

Further latent profile analysis revealed three distinct profiles of adherence to lymphedema prevention behaviors among patients after breast cancer surgery: Global Low-Adherence (13.4%), Moderate Adherence with Skin-Care Deficit (18.0%), and Active Prevention (68.6%), indicating substantial heterogeneity in prevention behavior patterns.

Patients in the Active Prevention profile accounted for the largest proportion (68.6%), suggesting that most patients were able to implement lymphedema prevention behaviors relatively well. This finding may be related to the increasing emphasis on comprehensive breast cancer management in recent years. Lymphedema risk management has gradually become incorporated into perioperative care and follow-up management, providing patients with more opportunities to acquire disease-related knowledge and health education. Consequently, greater awareness of the consequences of lymphedema and the benefits of prevention behaviors may be associated with the higher adherence observed in this profile (29). For these patients, regular follow-up and continuous health education remain important components of ongoing lymphedema risk management and may be relevant to sustained adherence over time.

The Moderate Adherence with Skin-Care Deficit profile accounted for 18.0% of the sample. Although these patients demonstrated relatively favorable overall adherence, skin-care behaviors were notably insufficient, which is consistent with previous findings (30, 31). One possible explanation is that the benefits of skin-care behaviors may not be immediately observable, which may be related to patients’ relatively lower prioritization of these behaviors. In contrast, behaviors such as functional exercise and protection of the affected limb may provide more direct and perceivable benefits, making them easier for patients to prioritize. Therefore, healthcare professionals should provide targeted education and skill-based guidance regarding skin care to enhance patients’ awareness of its importance and promote the comprehensive implementation of prevention behaviors.

The Global Low-Adherence profile included 13.4% of patients. Patients in this profile showed consistently low scores across all dimensions, reflecting an overall lack of adherence to lymphedema prevention behaviors. This pattern may be associated with lower educational attainment, limited health literacy, and insufficient disease-related knowledge. Patients with lower educational attainment and inadequate health literacy may have greater difficulties accessing and understanding information related to lymphedema risk and prevention strategies, thereby increasing the likelihood of insufficient adherence to prevention behaviors (27, 32). Therefore, greater attention should be directed toward this subgroup. Individualized health education, the use of visual materials, and family involvement may improve disease-related understanding and facilitate the implementation of prevention behaviors.

Overall, these findings highlight the importance of dynamically assessing adherence to lymphedema prevention behaviors among patients after breast cancer surgery. Implementing stratified management strategies based on distinct behavioral profiles may improve the targeting and effectiveness of health education and facilitate more individualized preventive care.

### 4.2 Factors associated with latent profiles of adherence to lymphedema prevention behaviors among patients after breast cancer surgery

Older age was associated with a lower likelihood of belonging to the Active Prevention profile, which is consistent with previous studies (33). With increasing age, patients may experience gradual declines in information acquisition ability, self-management capacity, and motivation to adopt health-related behaviors. Meanwhile, older patients are more likely to experience chronic comorbidities, reduced physical function, and increased family caregiving burdens. These characteristics may be accompanied by greater demands for managing existing health problems, which may be related to relatively less attention to potential lymphedema risks that have not yet occurred (34). In addition, older patients may experience greater difficulties in maintaining long-term prevention behaviors, such as protection of the affected limb, functional exercise, and self-monitoring, which may be associated with lower adherence to lymphedema prevention behaviors. Therefore, older patients may warrant greater attention in clinical care, particularly in relation to health education and continuous follow-up to support sustained behavioral implementation.

Patients with stage II breast cancer were more likely to belong to the Active Prevention profile. This finding may be related to differences in overall disease burden, physical function, treatment-related symptoms, and psychological distress across disease stages, which may be relevant to the implementation of lymphedema prevention behaviors (35). Perception of disease severity and health risks is an important prerequisite for the initiation of health behaviors (36). Compared with patients with stage I disease, those with stage II disease may have greater awareness of disease burden and potential risks, which may be associated with greater recognition of the importance of complication prevention. Thus, the combination of behavioral capacity and risk awareness may be related to the higher likelihood of active engagement in lymphedema prevention behaviors observed among patients with stage II disease. These findings suggest that, in clinical practice, patients with stage I disease may warrant greater attention to lymphedema risk education, particularly with regard to risk perception and awareness of prevention behaviors.

Patients who had not received chemotherapy or radiotherapy were more likely to belong to the Global Low-Adherence profile. Patients receiving adjuvant treatments, such as chemotherapy or radiotherapy, generally experience longer periods of healthcare contact and follow-up management. During this process, they may have more opportunities to receive lymphedema-related health education and risk information, which may be associated with greater awareness of disease risks and preventive strategies and with more sustained prevention behaviors. In contrast, patients who do not receive adjuvant therapy may have fewer opportunities for follow-up and health education, which may be associated with lower awareness of lymphedema risk and lower adherence to prevention behaviors. These findings suggest that healthcare professionals should not only focus on patients receiving comprehensive treatment but also strengthen ongoing health education and behavioral assessment among patients without adjuvant therapy exposure, thereby improving their awareness of lymphedema risk and long-term prevention motivation.

The present study found that higher levels of self-regulatory fatigue were associated with a lower likelihood of belonging to the Active Prevention profile, which is consistent with previous research findings (28). Self-regulatory fatigue refers to a state of depleted psychological resources resulting from sustained cognitive, emotional, and behavioral regulation during long-term disease management. According to the strength model of self-control, self-control is a limited resource, and continuous depletion of this resource may impair individuals’ ability to maintain health-related behaviors (37). Lymphedema prevention behaviors require long-term and continuous engagement, including protection of the affected limb, functional exercise, self-monitoring, and lifestyle management. The sustained effort required to perform these behaviors may place additional demands on psychological resources. Higher levels of self-regulatory fatigue may be associated with greater difficulties in implementing prevention behaviors and with a higher likelihood of insufficient adherence. Even when patients are aware of appropriate prevention behaviors, difficulties in behavioral execution may be present alongside a gap between knowledge and action, which may be related to less consistent maintenance of health behaviors. Therefore, healthcare professionals should consider assessing patients’ levels of self-regulatory fatigue, identifying individuals who experience difficulties in behavioral implementation, and providing ongoing behavioral support. Given the diurnal depletion effect of self-control capacity (38), healthcare providers may consider developing time-based management strategies according to patients’ daily routines. For example, behaviors requiring greater behavioral effort, such as functional exercise of the affected limb and self-monitoring, could be scheduled during periods when patients have higher energy levels, which may be more compatible with their daily behavioral capacity. In addition, goal decomposition strategies may be considered to divide complex lymphedema prevention behaviors into smaller, achievable short-term goals, which may make these behaviors more manageable for patients experiencing higher self-regulatory fatigue. For patients with higher levels of self-regulatory fatigue, healthcare professionals may consider incorporating psychological resource assessment into routine follow-up care and identifying difficulties in behavioral implementation at an early stage. Strategies such as positive feedback and motivational interviewing may also be considered as forms of behavioral support for this subgroup.

Higher levels of interoceptive sensitivity were associated with a greater likelihood of belonging to the Active Prevention profile. Interoceptive sensitivity refers to an individual’s ability to perceive, interpret, and integrate internal physiological signals. It not only reflects bodily awareness but also plays an important role in the initiation and maintenance of health behaviors (39, 40). Early manifestations of breast cancer-related lymphedema (BCRL) often include nonspecific symptoms such as limb heaviness, tightness, and mild swelling. Patients with higher interoceptive sensitivity may be more attentive to subtle bodily changes and potential risk signals, which may be associated with greater health awareness and more active engagement in lymphedema prevention behaviors. Conversely, lower interoceptive sensitivity may be associated with less attention to abnormal bodily signals and delayed recognition of symptoms or related behavioral responses. Therefore, interoceptive sensitivity may serve as an important assessment indicator in postoperative behavioral management among patients with breast cancer. Future rehabilitation programs may consider incorporating interventions such as mindfulness-based training and body awareness exercises to enhance patients’ attention to and utilization of bodily signals, strengthen proactive health management awareness, and facilitate the initiation and maintenance of lymphedema prevention behaviors.

## 5 Limitations

This study has several limitations. First, the single-center setting and convenience sampling may limit the generalizability of the findings. Participants were recruited from a tertiary hospital in Zhengzhou, China, and may differ from patients receiving care in other regions, healthcare settings, or healthcare systems. Patients with lower engagement in postoperative follow-up or more limited access to healthcare resources may also be underrepresented. Future multicenter studies involving more diverse populations and representative sampling methods are warranted to validate these profiles and their associated factors. Second, the cross-sectional design precludes conclusions regarding causality or temporal dynamics. Although several demographic, clinical, and psychosocial factors were associated with different adherence profiles, the temporal sequence between these factors and adherence behaviors could not be established. It also remains unclear whether patients maintain the same adherence profile over time or transition between profiles during postoperative recovery. Future longitudinal studies are needed to examine the stability and evolution of adherence profiles and to clarify temporal relationships between potential influencing factors and prevention behaviors. Third, the present study did not include all potentially relevant factors associated with heterogeneity in adherence behaviors. In particular, the time elapsed since breast cancer surgery was not specifically examined, and other psychosocial and healthcare-related factors may also be relevant to patients’ prevention behaviors. The omission of these variables may have limited the comprehensiveness of the profile characterization. Future studies should incorporate a broader range of clinical, psychosocial, behavioral, and healthcare-related factors to further characterize this heterogeneity. Finally, adherence was assessed using self-reported questionnaires, which may be subject to recall and social desirability biases. Self-reported behaviors may not fully correspond to patients’ actual behavioral performance. Future research combining self-reported measures with objective behavioral assessments and clinical indicators may provide a more comprehensive understanding of lymphedema prevention behaviors and their clinical relevance.

## 6 Conclusion

Adherence to lymphedema prevention behaviors among patients after breast cancer surgery demonstrated substantial heterogeneity, with latent profile membership associated with age, tumor stage, chemotherapy or radiotherapy experience, self-regulatory fatigue, and interoceptive sensitivity. These findings highlight the importance of recognizing individual differences in prevention behavior patterns and may inform stratified assessment and individualized preventive care. Healthcare professionals may consider patients’ psychological resources and interoceptive sensitivity when assessing adherence patterns and planning preventive care. This study provides new insights into behavioral heterogeneity among patients after breast cancer surgery and may contribute to the development of individualized prevention and rehabilitation approaches.

## Data Availability

The raw data supporting the conclusions of this article will be made available by the authors, without undue reservation.

## References

[1] Bray F Laversanne M Sung H Ferlay J Siegel RL Soerjomataram I . Global cancer statistics 2022: GLOBOCAN estimates of incidence and mortality worldwide for 36 cancers in 185 countries. CA Cancer J Clin. (2024) 74:229–63. doi: 10.3322/caac.21834, 38572751

[2] Giaquinto AN Sung H Newman LA Freedman RA Smith RA Star J . Breast cancer statistics 2024. CA Cancer J Clin. (2024) 74:477–95. doi: 10.3322/caac.21863, 39352042

[3] Feng M Zong N Cao M Xu H. Current cancer situation in China: good or bad news from the 2018 global Cancer statistics? Cancer Commun. (2019) 39, 1–12. doi: 10.1186/s40880-019-0368-6, 31030667 PMC6487510

[4] Tao X Li T Gandomkar Z Brennan PC Reed WM. Incidence, mortality, survival, and disease burden of breast cancer in China compared to other developed countries. Asia Pac J Clin Oncol. (2023) 19:645–54. doi: 10.1111/ajco.13958, 37026375

[5] Marco E Trépanier G Chang E Mauti E Jones JM Zhong T . Postmastectomy functional impairments. Curr Oncol. (2023) 25:1445–53. doi: 10.1007/s11912-023-01474-6 PMC10728246 37955831

[6] Thomas M. Breast cancer-related upper limb lymphoedema: an overview. Br J Community Nurs. (2021) 26:S30–5. doi: 10.12968/bjcn.2021.26, 33797944

[7] Armer J Fu MR. Age differences in post-breast cancer lymphedema signs and symptoms. Cancer Nurs. (2005) 28:200–7. doi: 10.1097/00002820-200505000-00007, 15915063

[8] Shen A Wu P Qiang W Fu X Zhu F Pang L . Factors associated with lymphedema self-management behaviours among breast cancer survivors: a cross-sectional study. J Clin Nurs. (2023) 32:7330–45. doi: 10.1111/jocn.16833, 37491890

[9] Donahue PMC MacKenzie A Filipovic A Koelmeyer L. Advances in the prevention and treatment of breast cancer-related lymphedema. Breast Cancer Res Treat. (2023) 200:1–14. doi: 10.1007/s10549-023-06947-7, 37103598 PMC10224871

[10] Nguyen TT Hoskin TL Habermann EB Cheville AL Boughey JC. Breast cancer-related lymphedema risk is related to multidisciplinary treatment and not surgery alone: results from a large cohort study. Ann Surg Oncol. (2017) 24:2972–80. doi: 10.1245/s10434-017-5960-x, 28766228 PMC5737818

[11] Cao M Wang Y Li J Li X Wang Z. Current status and influencing factors of adherence to lymphedema prevention behaviors among postoperative breast cancer patients: a scoping review. Chin J Mod Nurs. (2025) 31:4743–9. doi: 10.3760/cma.j.cn115682-20250107-00103

[12] Zhang Y Li N Chen J Luo X Li M Yan J. Breast cancer-related lymphedema risk-management behaviors among Chinese breast cancer survivors and relationships with socio-demographic and clinical characteristics: a longitudinal study. Patient Prefer Adherence. (2022) 16:797–808. doi: 10.2147/PPA.S356750, 35370404 PMC8965334

[13] Shen A Wu P Qiang W Zhu F Zhang Z Wang Y . Breast cancer survivors’ experiences of barriers and facilitators to lymphedema self-management behaviors: a theory-based qualitative study. J Cancer Surviv. (2025) 19:642–58. doi: 10.1007/s11764-023-01497-9, 37971555

[14] Cal A Bahar Z. Women’s barriers to prevention of lymphedema after breast surgery and home care needs: a qualitative study. Cancer Nurs. (2016) 39:E17–25. doi: 10.1097/NCC.0000000000000326, 26641643

[15] Ridner SH Bonner CM Deng J Sinclair VG. Voices from the shadows: living with lymphedema. Cancer Nurs. (2012) 35:E18–26. doi: 10.1097/NCC.0b013e31821404c0, 21558848 PMC3172392

[16] Silva-Smith AL Hanson CL Neubeck L Rowat A McHale S. Physical activity interventions framed by the health action process approach for adults with long-term conditions: a scoping review. IntJ Behav Med. (2024) 31:987–1017. doi: 10.1007/s12529-024-10305-2, 39009797 PMC11588932

[17] Lee CS Chu SH Dunne J Spintzyk E Locatelli G Babicheva V . Body listening in the link between symptoms and self-care management in cardiovascular disease: a cross-sectional correlational descriptive study. Int J Nurs Stud. (2024) 156:104809. doi: 10.1016/j.ijnurstu.2024.104809, 38788262

[18] Breast Cancer Professional Committee of Chinese Anti-Cancer Association Breast Oncology Group of Oncology Branch of Chinese Medical Association Shao Z Wu J Zhang J. Guidelines and specifications for diagnosis and treatment of breast cancer by the Chinese anti-Cancer association (2026 edition). China Oncol. (2025) 35:1157–255. doi: 10.19401/j.cnki.1007-3639.2025.12.009

[19] Zhang A Duan S. Mediating effect of social alienation on the relationship between fear of cancer recurrence and sleep quality among breast cancer patients during rehabilitation. J Nurs. (2022) 29:66–70. doi: 10.16460/j.issn1008-9969.2022.12.066

[20] Tang Y Yang D Zhang S Qin Y Xue J Jiang X. A latent profile analysis of patient activation in postoperative breast cancer patients: a cross-sectional study. Support Care Cancer. (2026) 34:156. doi: 10.1007/s00520-026-10397-4, 41627509

[21] Qiu X Wang Y Yao L . Development and application of the adherence to lymphedema prevention behaviors scale for breast cancer survivors during rehabilitation. Chin J Nurs Educ. (2025) 22:1006–13. doi: 10.3761/j.issn.1672-9234.2025.08.018

[22] Mehling WE Acree M Stewart A Silas J Jones A. The multidimensional assessment of interoceptive awareness, version 2 (MAIA-2). PLoS One. (2018) 13:e0208034. doi: 10.1371/journal.pone.0208034, 30513087 PMC6279042

[23] Teng B Wang D Su C Zhou H Wang T Mehling WE . The multidimensional assessment of interoceptive awareness, version 2: translation and psychometric properties of the Chinese version. Front Psych. (2022) 13:970982. doi: 10.3389/fpsyt.2022.970982, 36440402 PMC9691670

[24] Nes LS Ehlers SL Whipple MO Vincent A. Self-regulatory fatigue in chronic multisymptom illnesses: scale development, fatigue, and self-control. J Pain Res. (2013) 6:181–8. doi: 10.2147/JPR.S40014, 23526193 PMC3596127

[25] Wang L Zhang J Wang J Tao T Fan C Gao W . Reliability and validity of the Chinese version of the self-regulatory fatigue scale in young adults. Chin Ment Health J. (2015) 29:290–4. doi: 10.3969/j.issn.1000-6729.2015.04.010

[26] Wu X Jiang W Yao X . A qualitative study of facilitators and barriers to upper-limb functional exercise after discharge among patients following breast cancer surgery. Chinese General Practice Nursing. (2024) 22:550–4. doi: 10.12104/j.issn.1674-4748.2024.03.039

[27] Uhlmann RA Mott SL Curry M Phadke S Sugg SL Erdahl LM . Analysis of the understanding and worry about lymphedema of patients with breast cancer. Ann Surg Oncol. (2022) 29:6428–37. doi: 10.1245/s10434-022-12189-6, 35913669

[28] Sherman KA Koelmeyer L. Psychosocial predictors of adherence to lymphedema risk minimization guidelines among women with breast cancer. Psycho-Oncology. (2013) 22:1120–6. doi: 10.1002/pon.3111, 22689156

[29] Ridner SH Dietrich MS Kidd N. Breast cancer treatment-related lymphedema self-care: education, practices, symptoms, and quality of life. Support Care Cancer. (2011) 19:631–7. doi: 10.1007/s00520-010-0870-5, 20393753

[30] Sherman KA Miller SM Roussi P Taylor A. Factors predicting adherence to risk management behaviors of women at increased risk for developing lymphedema. Support Care Cancer. (2015) 23:61–9. doi: 10.1007/s00520-014-2321-1, 24970542 PMC4270823

[31] Tsuchiya M Masujima M Kato T Ikeda S-i Shimizu C Kinoshita T . Knowledge, fatigue, and cognitive factors as predictors of lymphoedema risk-reduction behaviours in women with cancer. Support Care Cancer. (2019) 27:547–55. doi: 10.1007/s00520-018-4349-0, 30014192

[32] Jiang W Chen L. Analysis of the factors and moderating role of self-care ability among patients with breast cancer-related lymphedema. J Clin Nurs. (2022) 32:926–40. doi: 10.1111/jocn.16495, 36101935

[33] Benzo MV Novotny P Benzo RP. Adding granularity of COPD self-management to impact quality of life. Chronic Obstr Pulm Dis. (2022) 9:277–84. doi: 10.15326/jcopdf.2021.0277, 35259291 PMC9166338

[34] Wu M Lin X Zhou C Liu W. Latent profile analysis of self-management activation among patients with renal calculi. Shenzhen J Integr Tradit Chin West Med. (2026) 36:21–5. doi: 10.16458/j.cnki.1007-0893.2026.04.006

[35] Yang S Chen L Guo G Chu H Kong D Jin J . Latent profile analysis of psychosocial adjustment among patients with breast cancer undergoing chemotherapy and its effect on social participation. J Shandong First Med Univ Shandong Acad Med Sci. (2026) 47:31–8. doi: 10.3969/j.issn.2097-0005.2026.01.005

[36] Yirsaw AN Tefera M Bogale EK Anagaw TF Tiruneh MG Fenta ET . Applying the health belief model to cervical cancer screening uptake among women in Ethiopia: a systematic review and meta-analysis. BMC Cancer. (2024) 24:1294. doi: 10.1186/s12885-024-13055-2, 39427116 PMC11489999

[37] Hagger MS Wood C Stiff C Chatzisarantis NLD. Ego depletion and the strength model of self-control: a meta-analysis. Psychol Bull. (2010) 136:495–525. doi: 10.1037/a0019486, 20565167

[38] Wagner DD Altman M Boswell RG Kelley WM Heatherton TF. Self-regulatory depletion enhances neural responses to rewards and impairs top-down control. Psychol Sci. (2013) 24:2262–71. doi: 10.1177/0956797613492985, 24026225 PMC4151046

[39] Mehling WE Price C Daubenmier JJ Acree M Bartmess E Stewart A. The multidimensional assessment of interoceptive awareness (MAIA). PLoS One. (2012) 7:e48230. doi: 10.1371/journal.pone.0048230, 23133619 PMC3486814

[40] Khoury NM Lutz J Schuman-Olivier Z. Interoception in psychiatric disorders: a review of randomized controlled trials with interoception-based interventions. Harv Rev Psychiatry. (2018) 26:250–63. doi: 10.1097/HRP.0000000000000170, 30188337 PMC6129986

