# Supplementary material for: Latent profiles of adherence to lymphedema prevention behaviors and associated factors among breast cancer patients after surgery: a cross-sectional study
Source: Front Public Health. 2026 Sep 21;14:1972630. doi: 10.3389/fpubh.2026.1972630 (PMC13635202; doi:10.3389/fpubh.2026.1972630)
Supplement: Supplementary file 1 [file Table_1.docx]

# **Table 1 Model fit indices for latent profile models of adherence to lymphedema prevention behaviors among postoperative breast cancer patients (N=612)**

| **Profile** | **AIC** | **BIC** | **aBIC** | **LMRT *P*** | **BLRT *P*** | **Entropy** | **Class probability** |
| --- | --- | --- | --- | --- | --- | --- | --- |
| 1 | 15458.866 | 15578.118 | 15492.398 | - | - | - | - |
| 2 | 14482.766 | 14703.602 | 14544.863 | <0.001 | <0.001 | 0.986 | 0.329 / 0.671 |
| 3 | 13871.384 | 13972.969 | 13899.949 | <0.001 | <0.001 | 0.941 | 0.134 / 0.180 / 0.686 |
| 4 | 16748.959 | 16885.877 | 16787.459 | <0.001 | <0.001 | 0.893 | 0.096 / 0.349 / 0.210 / 0.345 |
| 5 | 16560.652 | 16732.905 | 16609.088 | <0.001 | <0.001 | 0.895 | 0.058 / 0.352 / 0.207 / 0.345 / 0.038 |

*Note: AIC, Akaike information criterion; BIC, Bayesian information criterion; aBIC, sample-size-adjusted BIC; LMRT, Lo-Mendell-Rubin likelihood ratio test; BLRT, bootstrapped likelihood ratio test.*
