# Supplementary material for: Latent profiles of adherence to lymphedema prevention behaviors and associated factors among breast cancer patients after surgery: a cross-sectional study
Source: Front Public Health. 2026 Sep 21;14:1972630. doi: 10.3389/fpubh.2026.1972630 (PMC13635202; doi:10.3389/fpubh.2026.1972630)
Supplement: Supplementary file 2 [file Table_2.docx]

**Table 2 General characteristics of participants and univariate analysis of latent profiles of adherence to lymphedema prevention behaviors**

| **Variable** | **Category** | **Total (n=612)** | **Global Low- Adherence (n=82)** | **Moderate Adherence with Skin-Care Deficit (n=110)** | **Active Prevention (n=420)** | **Test statistic** | ***P* value** |
| --- | --- | --- | --- | --- | --- | --- | --- |
| Age | <40 years | 254 (41.50) | 16 (6.30) | 35 (13.78) | 203 (79.92) | 31.226¹⁾ | <0.001 |
|  | 40–49 years | 175 (28.59) | 32 (18.29) | 31 (17.71) | 112 (64.00) |  |  |
|  | ≥50 years | 183 (29.90) | 34 (18.58) | 44 (24.04) | 105 (57.38) |  |  |
| Education level | Junior high school or below | 181 (29.58) | 35 (19.34) | 31 (17.13) | 115 (63.54) | 9.174¹⁾ | 0.056 |
|  | Technical secondary/senior high school | 213 (34.80) | 27 (12.68) | 40 (18.78) | 146 (68.54) |  |  |
|  | Junior college or above | 218 (35.62) | 20 (9.17) | 39 (17.89) | 159 (72.94) |  |  |
| Employment status | Farming | 236 (38.56) | 45 (19.07) | 48 (20.34) | 143 (60.59) | 22.358¹⁾ | <0.001 |
|  | Formally employed | 234 (38.24) | 21 (8.97) | 49 (20.94) | 164 (70.09) |  |  |
|  | Other | 142 (23.20) | 16 (11.27) | 13 (9.15) | 113 (79.58) |  |  |
| Medical insurance | New Rural Cooperative Medical Scheme | 244 (39.87) | 44 (18.03) | 43 (17.62) | 157 (64.34) | 28.076¹⁾ | <0.001 |
|  | Urban Resident Medical Insurance | 172 (28.10) | 20 (11.63) | 47 (27.33) | 105 (61.05) |  |  |
|  | Urban Employee Medical Insurance | 196 (32.03) | 18 (9.18) | 20 (10.20) | 158 (80.61) | 9.909¹⁾ | 0.042 |
| Average monthly per-capita income | <RMB 3000 | 247 (40.36) | 39 (15.79) | 55 (22.27) | 153 (61.94) |  |  |
|  | RMB 3000–5000 | 206 (33.66) | 24 (11.65) | 35 (16.99) | 147 (71.36) |  |  |
|  | >RMB 5000 | 159 (25.98) | 19 (11.95) | 20 (12.58) | 120 (75.47) |  |  |
| Tumor stage | Stage I | 121 (19.77) | 27 (22.31) | 10 (8.26) | 84 (69.42) | 162.681¹⁾ | <0.001 |
|  | Stage II | 337 (55.07) | 44 (13.06) | 21 (6.23) | 272 (80.71) |  |  |
|  | Stage III or above | 154 (25.16) | 11 (7.14) | 79 (51.30) | 64 (41.56) |  |  |
| Type of surgery | Radical mastectomy | 242 (39.54) | 26 (10.74) | 54 (22.31) | 162 (66.94) | 14.108¹⁾ | 0.007 |
|  | Modified radical mastectomy | 274 (44.77) | 34 (12.41) | 46 (16.79) | 194 (70.80) |  |  |
|  | Breast-conserving surgery | 96 (15.69) | 22 (22.92) | 10 (10.42) | 64 (66.67) |  |  |
| Axillary lymph node dissection | Yes | 348 (56.86) | 32 (9.20) | 80 (22.99) | 236 (67.82) | 22.002¹⁾ | <0.001 |
|  | No | 264 (43.14) | 50 (18.94) | 30 (11.36) | 184 (69.70) |  |  |
| Chemotherapy/radiotherapy history | Yes | 504 (82.35) | 56 (11.11) | 107 (21.23) | 341 (67.66) | 28.394¹⁾ | <0.001 |
|  | No | 108 (17.65) | 26 (24.07) | 3 (2.78) | 79 (73.15) |  |  |
| Affected limb is dominant hand | Yes | 339 (55.39) | 34 (10.03) | 52 (15.34) | 253 (74.63) | 13.365¹⁾ | 0.001 |
|  | No | 273 (44.61) | 48 (17.58) | 58 (21.25) | 167 (61.17) |  |  |
| Self-regulatory fatigue, median (IQR) |  | 29 (25, 44) | 49 (45, 51) | 42 (30, 49) | 27 (24, 30) | 165.635²⁾ | <0.001 |
| Interoceptive sensibility, median (IQR) |  | 105 (87, 110) | 84 (74, 88) | 86 (84, 105) | 107 (102, 112) | 164.332²⁾ | <0.001 |

*Note: 1) χ² test; 2) Kruskal-Wallis H test. Data are n (%) unless otherwise noted.*
