# Supplementary material for: Latent profiles of adherence to lymphedema prevention behaviors and associated factors among breast cancer patients after surgery: a cross-sectional study
Source: Front Public Health. 2026 Sep 21;14:1972630. doi: 10.3389/fpubh.2026.1972630 (PMC13635202; doi:10.3389/fpubh.2026.1972630)
Supplement: Supplementary file 3 [file Table_3.docx]

**Table 3 Multinomial logistic regression analysis of latent profile membership (reference: Global Low-Adherence)**

| **Variable** | ***β*** | ***SE*** | ***P* value** | ***OR*** | ***95% CI*** |
| --- | --- | --- | --- | --- | --- |
| **Moderate Adherence with Skin-Care Deficit (vs. Global Low-Adherence)** | | | | | |
| Constant | 1.635 | 2.819 | 0.562 | - | - |
| Age (40–49 years) | -1.244 | 0.563 | 0.027 | 0.288 | (0.10, 0.87) |
| Tumor stage (Stage II) | 1.142 | 0.569 | 0.045 | 3.314 | (1.03, 9.55) |
| Chemotherapy/radiotherapy history (No) | -2.704 | 0.785 | <0.001 | 0.067 | (0.01, 0.31) |
| **Active Prevention (vs. Global Low-Adherence)** | | | | | |
| Constant | 3.940 | 2.686 | <0.001 | - | - |
| Age (40–49 years) | -2.718 | 0.511 | <0.001 | 0.066 | (0.02, 0.18) |
| Age (≥50 years) | -2.256 | 0.476 | <0.001 | 0.105 | (0.04, 0.27) |
| Tumor stage (Stage II) | 1.323 | 0.432 | 0.002 | 3.755 | (1.61, 8.75) |
| Self-regulatory fatigue | -0.174 | 0.028 | <0.001 | 0.840 | (0.80, 0.89) |
| Interoceptive sensibility | 0.040 | 0.019 | 0.036 | 1.041 | (1.00, 1.08) |

*Note: SE, standard error; OR, odds ratio; CI, confidence interval.*
